# LONGITUDINAL CHARACTERISTICS OF INFORMAL CAREGIVERS OF COMMUNITY-DWELLING PERSONS WITH DEMENTIA

**DOI:** 10.1093/geroni/igz038.1688

**Published:** 2019-11-08

**Authors:** Eric Jutkowitz, Joseph E Gaugler, Amal Trivedi, Lauren L Mitchell, Pedro L Gozalo

**Affiliations:** 1 Brown University, Providence, Rhode Island, United States; 2 University of Minnesota - School of Public Health, Division of Health Policy and Management, Minneapolis, Minnesota, United States; 3 Minneapolis VA Health Care System, Minneapolis, Minnesota, United States

## Abstract

Alzheimer’s disease and related dementias (ADRD) affect >5 million Americans. Persons with ADRD experience functional limitations and often require support from informal caregivers (e.g., help with feeding). Little is known about how caregiving evolves over the full course of the disease. We used data from the Health and Retirement Study to identify incident predicted community-dwelling persons with ADRD (n=565), their informal caregivers, and the total hours of care they received in a month from predicted incidence up to 8-years post incidence. We estimated linear mixed-effects models to determine the characteristics of the person with ADRD that are associated with trajectories of caregiving hours. At predicted incidence, persons with ADRD received 120 hours of care in a month of which spouses provided 30% of care hours, adult children provided 32% of care hours, other relatives provided 12% of care hours, and non-relatives (including paid support) provided 25% of care hours. By 8-years post incidence, persons with ADRD still in the community (n=23) received 303 hours of care in a month of which spouses provided 28% of care hours, adult children provided 41% of care hours, other relatives provided 3% of care hours, and non-relatives provided 28% of care hours. Having great grandchildren and more functional limitations were associated with receiving more hours of informal care. Attrition (mortality and residing in a nursing home) was influenced by hours of care received in the previous interview and resulted in those that remained in the community being persons that required less care.

